# Integrated Tuberculosis and COVID-19 Activities in Karachi and Tuberculosis Case Notifications

**DOI:** 10.3390/tropicalmed7010012

**Published:** 2022-01-15

**Authors:** Amyn A. Malik, Hamidah Hussain, Rabia Maniar, Nauman Safdar, Amal Mohiuddin, Najam Riaz, Aneeta Pasha, Salman Khan, Syed Saleem Hasan Kazmi, Ershad Kazmi, Saira Khowaja

**Affiliations:** 1 Interactive Research and Development (IRD) Global, Singapore 238884, Singapore; hamidah.hussain@ird.global (H.H.); nauman.safdar@ird.global (N.S.); najam.riaz@ird.global (N.R.); saira.khowaja@ird.global (S.K.); 2 Yale Institute for Global Health, Yale University, New Haven, CT 06510, USA; 3 Department of Internal Medicine, Yale School of Medicine, Yale University, New Haven, CT 06510, USA; 4 Global Health Directorate, Indus Health Network, Karachi 75190, Pakistan; rabiaanism@gmail.com (R.M.); amal44@gmail.com (A.M.); 5 Interactive Research and Development (IRD) Pakistan, Karachi 75600, Pakistan; aneeta.pasha@ird.global; 6 Communicable Diseases Control, Department of Health, Government of Sindh, Hyderabad 65320, Pakistan; drsalmankhan@live.com (S.K.); saleemhk60@yahoo.co.uk (S.S.H.K.); ershadkazmi@gmail.com (E.K.)

**Keywords:** COVID-19, tuberculosis, screening, case notification, active case finding

## Abstract

As the COVID-19 pandemic surged, lockdowns led to the cancellation of essential health services. As part of our Zero TB activities in Karachi, we adapted our approach to integrate activities for TB and COVID-19 to decrease the impact on diagnosis and linkage to care for TB treatment. We implemented the following: (1) integrated COVID-19 screening and testing within existing TB program activities, along with the use of an artificial intelligence (AI) software reader on digital chest X-rays; (2) home delivery of medication; (3) use of telehealth and mental health counseling; (4) provision of PPE; (5) burnout monitoring of health workers; and (6) patient safety and disinfectant protocol. We used programmatic data for six districts of Karachi from January 2018 to March 2021 to explore the time trends in case notifications, the impact of the COVID-19 pandemic, and service adaptations in the city. The case notifications in all six districts in Karachi were over 80% of the trend-adjusted expected notifications with three districts having over 90% of the expected case notifications. Overall, Karachi reached 90% of the expected case notifications during the COVID-19 pandemic. The collaborative efforts by the provincial TB program and private sector partners facilitated this reduced loss in case notifications.

## 1. Introduction

The COVID-19 pandemic has resulted in approximately 311 million patients and 5.5 million deaths globally as of 11 January 2022 [1]. As countries scramble to control the pandemic and institute various levels of lockdowns to prevent the spread, there are major implications for health system and essential services. Experience from previous outbreaks has shown that there is a decrease in the uptake and utilization of health services [2]. Tuberculosis (TB) and COVID-19 have the same mode of transmission, where similar socio-economic determinants with poorer and marginalized communities are worse off [3]. Co-infection with SARS-CoV-2 in a TB patient may lead to immune cell anergy and precipitate respiratory failure [4]. The situation has been further exacerbated with unequitable distribution and access to health services and vaccines. The rapid increase in COVID-19 cases has led to TB services being severely impacted due to the cancellation of essential health services in high burden TB countries [5,6,7]. The pivoting of health services infrastructure and workforce towards COVID-19 has not helped either [5,8]. This has led to increased diagnostic delays and patients presenting with severe TB disease [9]. The WHO Global TB Report 2021 estimated that 1.3 million (18%) fewer people received TB care in 2020 compared to 2019 and this led to increased mortality due to the disease [10]. Unless the situation improves and additional measures are considered, it is estimated that an additional 6.3 million TB patients and 1.4 million TB deaths will be reported between 2020 and 2025 [11].

Pakistan is a high TB burden country with an annual TB incidence of 259/100,000 [10]. Pakistan’s first COVID-19 patient was in February 2020, which resulted in complete and subsequent waves of smart lockdowns [12,13]. This resulted in the closure of many outpatient departments leading to a 45% decrease in case notification for drug-susceptible TB patients between February and April 2020 [14]. As part of the Zero TB work in Karachi [15], Interactive Research and Development (IRD) as technical partner in collaboration with the Global Health Directorate, Indus Health Network operated TB services across multiple sites. Karachi is administratively divided into six districts (a seventh district has been added since the time of data collection and analysis). Pre-pandemic, sites operated by the team contributed approximately 80% of the total case notifications in the city. With the closure of sites, the Zero TB Karachi team had to adapt in real time to continue to provide TB services and to diagnose and link patients to care with COVID-19. 

Here, we describe in detail the adaptations made in the time of COVID-19 and analyze programmatic data reported to the Department of Health, Government of Sindh from six districts of Karachi to understand the impact of these adaptations on TB case notifications. In the discussion, we present and elaborate on the lessons learned during the adaptations made to improve TB care. 

## 2. Methods

### 2.1. Adaptations

#### 2.1.1. Integrating TB and COVID-19 Activities

TB and COVID-19 share the same clinical features, which provided an opportunity for co-screening. In March 2020, we integrated COVID-19 screening and testing into our existing TB services. We piloted at a hospital where verbal symptom screen along with digital chest X-ray with an artificial intelligence (AI) software was used to co-screen for both TB and COVID-19 [8,16]. Later, co-screening was extended to community settings using a mobile van with walk-through and drive-through TB and COVID-19 screening at hot spots. Verbal symptom screen included symptoms reported for COVID-19 and TB including fever, cough, shortness of breath, fatigue, diarrhea, body/headache, loss of sense of smell or taste, sore throat, chills, chest pain, and other flu-like symptoms.

We also integrated TB contact screening with activities to contact trace people exposed to SARS-CoV-2. Community health workers (CHWs) are already experienced in contact tracing activities for TB, hence it allowed for a quick integration and mobilization of combined contact tracing activities.

#### 2.1.2. Delivery of Medicines at Doorsteps

As lockdown was instituted throughout the city, and hospitals closed out-patient clinics, TB patients and household contacts on preventive treatment were unable to travel to clinic to pick up their medications. To avoid disruption in treatment, we delivered all TB medication to patients’ home, including preventive treatment medicines. We also increased the number of doses that were given at any visit from 1 month to 2–3 months. It ensured better logistical coordination and decreased the patient–healthcare provider interaction to reduce risk of COVID-19 transmission.

#### 2.1.3. Personal Protection Equipment (PPE) Provision

To allow complete safety of healthcare providers deployed at these sites, uninterrupted provision of essential personal protective equipment (PPE) was ensured. Teams were trained on the importance of adequate PPE, maintenance of essential infection control practices, along with donning and doffing techniques and waste disposal protocols.

#### 2.1.4. Patient Safety and Disinfection Protocol

**Figure 1:**
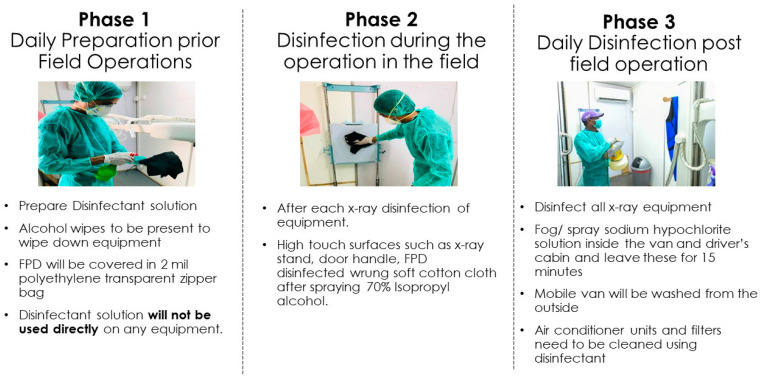
Mobile X-ray van disinfection protocol to ensure patient safety.

A comprehensive protocol was established to limit the spread of infection in mobile X-ray vans for integrated screening. A daily process of phasic infection control activities prior, during, and post-screening was employed to ensure complete disinfection of all surfaces, spaces, and equipment (Figure 1).

#### 2.1.5. Workforce Burnout Monitoring

Healthcare providers from various institutions across Karachi and Sindh were also provided with well-being check ins and counseling at regular intervals by our mental health team to monitor for burnout and depression. Screening for depression and anxiety was conducted using standardized scales (PHQ9 and GAD7) [17,18].

#### 2.1.6. Use of Telehealth and Mental Health Integration

Patients testing positive at our lab were followed up using telephone calls to ensure proper care, including mental and emotional well-being check-ins by our mental health team [19]. We also integrated mental health screening for depression and anxiety at these health encounters. As the volume of patients increased, the telehealth services were integrated in a TB call center that was already operational.

The mental health team called patients and community members tested for COVID-19 to provide emotional and mental health support and guidance on quarantine, adherence counseling, contact tracing, de-stigmatization of COVID-19, and referrals to essential services. Screening for depression and anxiety was conducted using standardized scales (PHQ9 and GAD7) [17,18].

### 2.2. Programmatic Data

We extracted programmatic data for 6 districts of Karachi (South, Central, Korangi, Malir, East, and West) from January 2018 to March 2021 from Department of Health, Government of Sindh to explore the time trends in case notification, the impact of COVID-19 pandemic, and service adaptations in the city. 

### 2.3. Data Analysis

We generated trend-corrected expected case notifications quarterly for Karachi from Q1, 2020, to Q1, 2021, using data trends from 2018 and 2019 for all case notifications and case notifications for children separately (extrapolating from a regression model). We then compared the actual reported case notifications to these expected case notification numbers to understand the impact. We also compared the male to female ratio across the 6 districts to understand gender-related effects of the pandemic and service adaptations.

### 2.4. Ethical Approval

As the study used aggregated programmatic data that are routinely reported, an ethical approval was not required. 

## 3. Results

**Figure 2:**
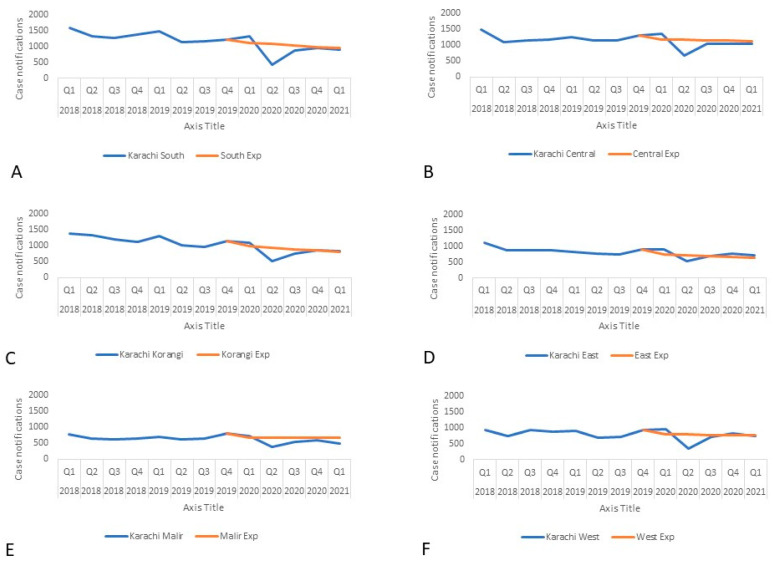
Actual and expected TB case notifications in Karachi by district from 2018–2021: (**A**) District South, (**B**) District Central, (**C**) District Korangi, (**D**) District East, (**E**) District Malir, (**F**) District West.

Between January 2020 and March 2021, the case notifications in all six districts in Karachi were over 80% of the trend-adjusted expected notifications with three districts, Korangi, East, and West, having over 90% of the expected case notifications (Figure 2). This translated into an average of 90% across Karachi. There was an initial dip in case notifications in all districts in Q2, 2020, but as TB activities were integrated with COVID-19 work, there was a sustained increase throughout the study period.

**Figure 3:**
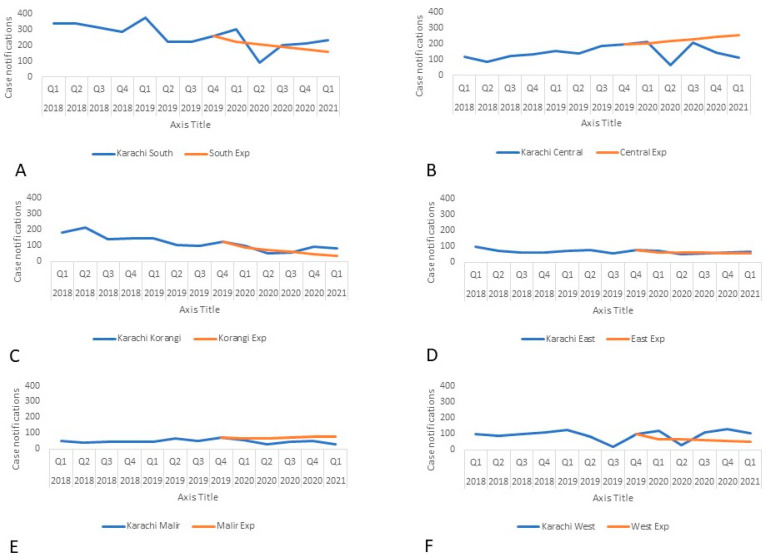
Actual and expected pediatric TB case notifications in Karachi by district from 2018–2021: (**A**) District South, (**B**) District Central, (**C**) District Korangi, (**D**) District East, (**E**) District Malir, (**F**) District West.

**Figure 4:**
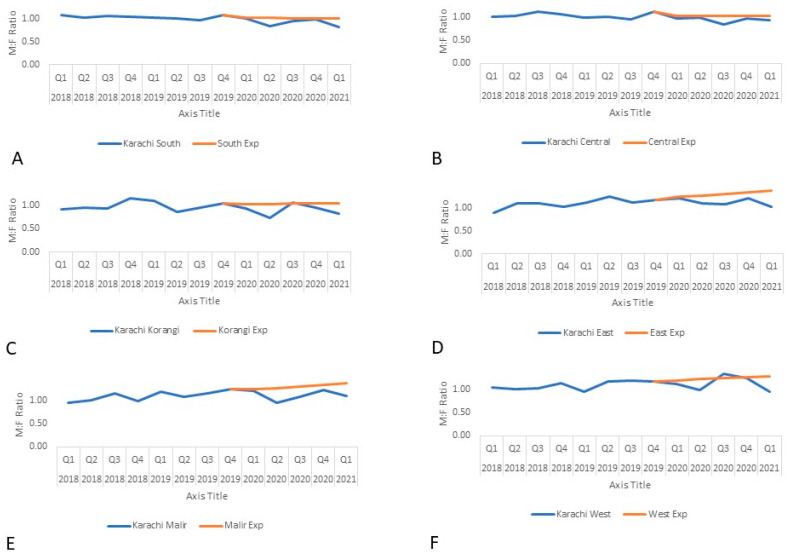
Actual and expected male to female ratio of TB case notifications in Karachi by district from 2018–2021: (**A**) District South, (**B**) District Central, (**C**) District Korangi, (**D**) District East, (**E**) District Malir, (**F**) District West.

The average pediatric case notification across Karachi was 94% of the trend-adjusted expected notifications with four of the six districts, South, Korangi, East, and West, exceeding 100% (Figure 3). The male to female ratio was lower in all six districts of Karachi (Figure 4) with Korangi, East, and Malir being less than 90% of the expected notifications. 

The share of private sector case notifications was 65% on average between 2020 and 2021 compared to 60% between 2018 and 2019. 

## 4. Discussion

Our results show that, overall, Karachi reached 90% of the expected case notifications during the COVID-19 pandemic. The collaborative efforts by the PTP Sindh and private sector partners facilitated this reduced loss in case notification. Our findings support calls for an integrated comprehensive approach to care delivery for TB and COVID-19 [6,7]. 

Globally, it is estimated that there was a 18% reduction in the number of reported cases in 2020 as compared to 2019 [10] ranging from 16–41% in 9 high-burden countries including Pakistan [20]. A recent Global Fund report estimated that there was a decrease of about 1 million in the number of people tested and treated for TB between 2019 and 2020 (18%), with Pakistan accounting for approximately 50,000 (15% drop) less people tested and treated for TB during this period. It is important to note that in Q2, 2020, there was a sharp drop as documented earlier [14]. This was the time when stay at home orders were in place and the TB community was evaluating different adaptations. We see an increase in case notifications from Q3, 2020, that continues until Q1, 2021, after the implementation of outreach activities. 

We saw higher than expected pediatric case notifications. Annually, 2.9 million people with incident TB disease are not diagnosed [10]. The gap between incidence and diagnosis is especially large for children [21]. Increased screening efforts can help curtail this diagnosis gap [22,23,24]. It is also possible that parents worried about their child being sick with COVID-19 were more likely to get screened during the co-screening activities conducted as part of the adaptation. 

During the COVID-19 pandemic, many private providers continued to provide TB services, even when public sector hospitals had reduced access [25]. Our results are reflective of health systems access during the COVID-19 times given the availability of services and as perhaps people may not be accessing larger public health facilities. 

A major limitation of our study is that we use programmatic reporting data aggregated at the district level and hence are not able to show the direct effect of increased screening. However, the increased effort resulting in reduced actual to expected case notification gap in some districts show a dose–response gradient. The strengths of our study include use of routinely collected surveillance data that capture changes in case notifications directly. 

### Lessons Learned

It was essential to be adaptive of how services were provided, but an integral component was listening to the various stakeholders to understand their challenges and needs. The stigma and fear in general population of contracting COVID-19 was important to understand when selecting locations where TB services could be delivered. The lack of resources in public sector to tackle COVID-19 and the slowness of the system to be adaptive were considered as we proposed the integration of services within public sector hospital and community spaces. While it was important to maintain TB services, we had to be aware of the community and other partner demands in a pandemic environment and leveraged existing resources as an opportunity to maintain TB services.

Stigma and fear were not limited to the patients/general population but rampant within the front-line workers as well. The pandemic was new for them, and they did not want to bring the virus into their home and infect their families. Since we were requesting our healthcare workers to continue activities, it was realized that a positive safety climate had to be established for our staff to give them the assurance that their and their families’ well-being was a priority [26], as they continued to work while the rest of the country was in lockdown. The availability of human resources is critical to continuing essential services.

It is important to note that while human resources were integrated for both TB and COVID-19, the digital tools such as artificial intelligence software were also deployed and integrated allowing for higher throughput. This integration of human resource and digital tools may have decreased the overall cost of running an integrated program. There was also a need for task shifting as we adapted practices and training of TB health workers on nasopharyngeal swab collection for COVID-19 testing. TB laboratory staff were also trained for SARS-CoV-2 testing. 

While globally directly observed therapy (DOT) has been an inherent part of TB, in our setting, the majority of the patients would (pre-pandemic) come to centers to collect their medication. It took significant effort and coordination to help deliver medication to patients’ homes. Alternately, patients were called in less frequently, and medicines were dispensed for a longer duration. There is a need to build a more resilient and decentralized system of TB treatment and medicine delivery, such as diagnostics of TB so that these services are not impacted or come to a halt during the pandemic and other emergencies. 

TB programs in low- and middle-income countries are dependent on funding from multi-lateral organizations, and their ability to be flexible in the utilization of funds for locally driven solutions is critical. The response to the pandemic required that we adapt quickly based on the local context and required the funding organizations to allow flexibility in fund utilization. There is also a need to increase the share of TB domestic funding to allow implementers more flexibility in adapting quickly and without bureaucratic hurdles [27]. 

## 5. Conclusions

Our results show that increased integration of TB and COVID-19-related activities and adaptations can limit the impact of the COVID-19 pandemic on TB case notifications and services and decrease the gap between expected and actual notifications. Public Health programs globally need to reevaluate their strategies for delivering healthcare. To avoid the indirect consequences of COVID-19 on TB, business as usual is not an option. There is a need for an integrated, adaptive, and comprehensive approach to health delivery. 

## Data Availability

All required data is present in the manuscript.
