# Correction: Malik et al. Integrated Tuberculosis and COVID-19 Activities in Karachi and Tuberculosis Case Notifications. *Trop. Med. Infect. Dis.* 2022, *7*, 12

**DOI:** 10.3390/tropicalmed7050063

**Published:** 2022-04-21

**Authors:** Amyn A. Malik, Hamidah Hussain, Rabia Maniar, Nauman Safdar, Amal Mohiuddin, Najam Riaz, Aneeta Pasha, Salman Khan, Syed Saleem Hasan Kazmi, Ershad Kazmi, Saira Khowaja

**Affiliations:** 1 Interactive Research and Development (IRD) Global, Singapore 238884, Singapore; hamidah.hussain@ird.global (H.H.); nauman.safdar@ird.global (N.S.); najam.riaz@ird.global (N.R.); saira.khowaja@ird.global (S.K.); 2 Yale Institute for Global Health, Yale University, New Haven, CT 06510, USA; 3 Department of Internal Medicine, Yale School of Medicine, Yale University, New Haven, CT 06510, USA; 4 Global Health Directorate, Indus Health Network, Karachi 75190, Pakistan; rabiaanism@gmail.com (R.M.); amal44@gmail.com (A.M.); 5 Interactive Research and Development (IRD) Pakistan, Karachi 75600, Pakistan; aneeta.pasha@ird.global; 6 Communicable Diseases Control, Department of Health, Government of Sindh, Hyderabad 65320, Pakistan; drsalmankhan@live.com (S.K.); saleemhk60@yahoo.co.uk (S.S.H.K.); ershadkazmi@gmail.com (E.K.)

## Reference List Correction

The authors wish to revise the second citation of reference [26] to [27] in the original article main text [1] and add a new reference [27] to the list of references.

On page 7, the original sentences are as follows: The response to the pandemic required that we adapt quickly based on the local context and required the funding organizations to allow flexibility in fund utilization. There is also a need to increase the share of TB domestic funding to allow implementers more flexibility in adapting quickly and without bureaucratic hurdles [26].

The correction is as follows: The response to the pandemic required that we adapt quickly based on the local context and required the funding organizations to allow flexibility in fund utilization. There is also a need to increase the share of TB domestic funding to allow implementers more flexibility in adapting quickly and without bureaucratic hurdles [27].

27. Malik, A.A.; Hussain, H.; Creswell, J.; Siddiqui, S.; Ahmed, J.F.; Madhani, F.; Habib, A.; Khan, A.J.; Amanullah, F. The Impact of Funding on Childhood TB Case Detection in Pakistan. *Trop. Med. Infect. Dis.* **2019**, *4*, 146. https://doi.org/10.3390/tropicalmed4040146.

The authors regret any inconvenience caused by this correction and assure that the scientific conclusions are unaffected. The original article has been updated accordingly.
